# *MdATG18a* overexpression improves basal thermotolerance in transgenic apple by decreasing damage to chloroplasts

**DOI:** 10.1038/s41438-020-0243-2

**Published:** 2020-03-01

**Authors:** Liuqing Huo, Xun Sun, Zijian Guo, Xin Jia, Runmin Che, Yiming Sun, Yanfei Zhu, Ping Wang, Xiaoqing Gong, Fengwang Ma

**Affiliations:** 1 State Key Laboratory of Crop Stress Biology for Arid Areas/Shaanxi Key Laboratory of Apple, College of Horticulture, Northwest A&F University, Yangling, 712100 Shaanxi China; 2 Center of Pear Engineering Technology Research, State Key Laboratory of Crop Genetics and Germplasm Enhancement, College of Horticulture, Nanjing Agricultural University, Nanjing, 210095 China

**Keywords:** Heat, Photosynthesis

## Abstract

High temperature is an abiotic stress factor that threatens plant growth and development. Autophagy in response to heat stress involves the selective removal of heat-induced protein complexes. Previously, we showed that a crucial autophagy protein from apple, MdATG18a, has a positive effect on drought tolerance. In the present study, we treated transgenic apple (*Malus domestica*) plants overexpressing *MdATG18a* with high temperature and found that autophagy protected them from heat stress. Overexpression of *MdATG18a* in apple enhanced antioxidase activity and contributed to the production of increased beneficial antioxidants under heat stress. Transgenic apple plants exhibited higher photosynthetic capacity, as shown by the rate of CO_2_ assimilation, the maximum photochemical efficiency of photosystem II (PSII), the effective quantum yield, and the electron transport rates in photosystems I and II (PSI and PSII, respectively). We also detected elevated autophagic activity and reduced damage to chloroplasts in transgenic plants compared to WT plants. In addition, the transcriptional activities of several *HSP* genes were increased in transgenic apple plants. In summary, we propose that autophagy plays a critical role in basal thermotolerance in apple, primarily through a combination of enhanced antioxidant activity and reduced chloroplast damage.

## Introduction

As global warming intensifies, the average temperature of the earth is rising. Through climate prediction models, the Intergovernmental Panel on Climate Change predicted that global surface temperature will rise by 2.5–5.4 °C during the 21st century. An above optimal temperature is an abiotic stress factor that threatens plant growth and development. Heat shock can even cause morphological, physiological, and biochemical changes in plants, dramatically affecting plant growth and reproduction^1,2^. As sessile organisms, plants cannot escape continuous harsh environmental factors and are compelled to change their cellular state to cope with damage. Therefore, plants have evolved a variety of strategies to accommodate irregular increases in temperature. First, plants can survive when they are exposed directly to extremely high temperatures. This ability is known as basal thermotolerance^3,4^. Second, plants exhibit a significantly increased tolerance to fatal heat stress after pre-exposure to a nonlethal heat stimulus for a certain period. This heat adaptive response is called acquired thermotolerance^5^.

When plants encounter high temperatures, a series of damaging events, including the excessive production of reactive oxygen species (ROS), protein misfolding and denaturation, and degradation of cellular structural components, occur^6^. ROS, such as H_2_O_2_, O_2_^–^, -OH, and ^1^O_2_, induced by heat stress originate primarily from chloroplasts, mitochondria, peroxisomes, and the plasma membrane^7^. As ROS may cause damage to plants, plants have also developed a set of mechanisms to maintain proper cellular ROS levels through production and scavenging pathways^8–10^. Destruction of ROS scavenging systems led to the increased sensitivity of plants to heat stress, which suggested the importance of ROS regulatory systems in the protection of plants from heat stress^11,12^. For example, the absence of ascorbate peroxidase 1 (APX1), a cytoplasmic ROS-scavenging enzyme, led to high temperature intolerance and growth retardation^12^.

Notably, plant photosynthesis is an important physiological process that is hypersensitive to high temperatures at the subcellular level. Photosynthesis is often inhibited early following exposure to heat stress, and the photosynthetic apparatus is vulnerable to damage under high temperatures^13^. Because heat stress commonly causes severe thermal damage to PSII, it dramatically affects photosynthetic electron transfer and ATP synthesis^1,14^. In addition, exposure to high temperatures was found to alter the ultrastructure of chloroplasts and integrity of thylakoid membranes^1,15^, which significantly inhibited the thermotolerance of plants.

Therefore, the protection of chloroplasts under heat stress is essential for plants. The synthesis of heat shock proteins (Hsps) is an important response to heat stress in all organisms. HSPs play a major role in the protection of cellular protein components and act as chaperones by regulating the folding and accumulation of proteins or preventing irreversible protein aggregation^10^. HSP synthesis can be induced by mild heat priming or heat shock^16,17^.

Heat stress, especially extreme heat stress, triggers the misfolding and denaturation of proteins that are highly toxic because they can nonspecifically bind cellular components. Misfolded proteins cause the cytoplasmic protein response or unfolded protein response in the endoplasmic reticulum, which stimulates the occurrence of autophagy^18^. Autophagy is an evolutionarily conserved pathway for the degradation of unnecessary cytoplasmic constituents that ensures the circulation of cellular proteins. It plays an important role in nutrient cycling in plants and underpins plant tolerance to various biotic and abiotic stresses^19^. Heat stress significantly induced the expression of autophagosome-related (ATG) genes and accumulated autophagosomes in tomato. *Arabidopsis atg5* and *atg7* mutants displayed compromised heat tolerance compared with wild-type (WT) *Arabidopsis*, as shown by their increased morphological symptoms, which involved enhanced defects in photosynthetic efficiency and capacity after heat stress^20^. Similar results were described for *ATG5-* or *ATG7-*silenced tomato mutants exposed to heat stress^21^. These results indicated that autophagy plays an active role in the basal thermotolerance of plants. Interestingly, the acquired thermotolerance of *Arabidopsis atg* mutants was similar to that of WT plants^22^. Autophagy mutants even survived better under high temperature stress 4 days after priming heat treatment. This demonstrated that autophagy also affects heat tolerance by altering other proteins, such as HSPs.

Previously, we found that the autophagy-related gene *MdATG18a* in apple enhanced plant resistance to drought stress^23^, nitrogen depletion^24^, and the fungus *Diplocarpon mali*^25^. Here, we employed *MdATG18a*-overexpressing (OE) apple plants to unveil the function of this gene under extreme heat stress. In addition, the physiological mechanism of the heat resistance of apple has not been widely studied. In this study, we found that autophagy plays an important role in the heat resistance of apple. Through a series of studies, we found that *MdATG18a* improved thermotolerance by enhancing autophagic activity, protecting chloroplasts, maintaining higher levels of photosynthesis, scavenging toxic ROS, and inducing *HSP* expression in apple.

## Results

### Overexpression of *MdATG18a* led to enhanced heat tolerance in apple

**Fig. 1:**
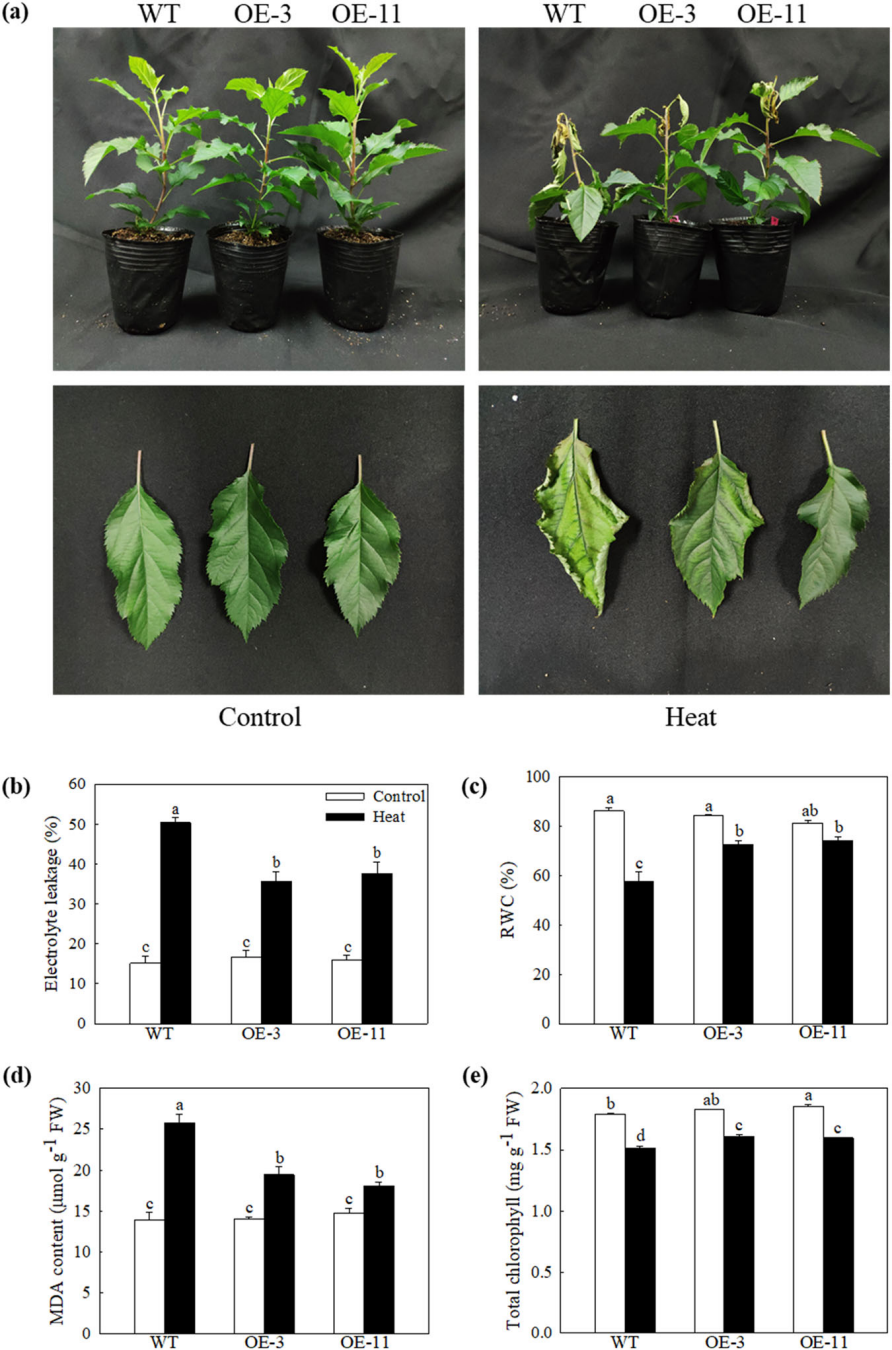
Overexpression of *MdATG18a* confers enhanced heat tolerance to transgenic apple plants. **a** Phenotypes of WT and *MdATG18a*-overexpressing apple plants under a normal temperature and after heat stress. **b** Electrolyte leakage, **c** relative water content (RWC), **d** malondialdehyde (MDA) concentration, and **e** chlorophyll concentration in WT and transgenic apples treated with or without high temperature. Data are shown as the means of three replicates with SEs. Different letters indicate significant differences between treatments, according to one-way ANOVA and Tukey’s multiple range test (*P* < 0.05).

Previously, we found that the *MdATG18a* transcript was significantly upregulated by heat stress^26^. To further investigate how *MdATG18a* functions under heat stress in apple, two previously obtained transgenic apple lines^23^ were used here. When exposed directly to 48 °C for 6 h, many of the leaves of the WT plants were burned and shriveled, but only the young leaves on the top of OE plants displayed symptoms of dehydration and burn, and the mature leaves remained green and vigorous (Fig. 1a). The detected REL was significantly increased due to injury but still much lower in the transgenic lines than in WT apple (Fig. 1b). The same result was observed when we measured the MDA concentration in different plants under heat stress (Fig. 1d), which showed mild damage to OE plants caused by high temperature. In addition, a high temperature led to wilting in leaves and reduced RWC, yet we found that wilting was obviously higher in OE lines than in WT plants (Fig. 1c). Total chlorophyll concentrations were decreased after heat treatment, but this reduction was much smaller in OE plants than in WT plants (Fig. 1e). All these results showed that heat stress decreased physiological damage to transgenic apple, which implies a positive role for *MdATG18a* in apple in response to heat stress.

### Overexpression of MdATG18a in apple enhanced the activities of ROS-scavenging enzymes and decreased ROS accumulation under heat stress

**Fig. 2:**
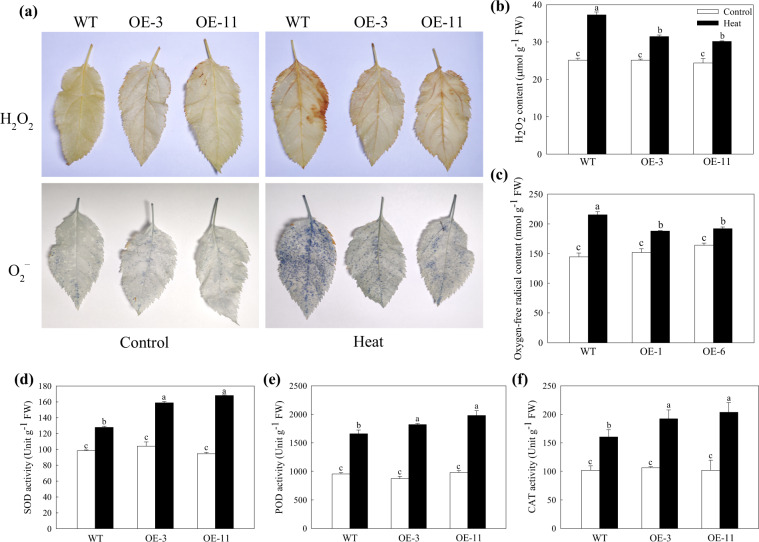
Changes in the levels of accumulated ROS and activities of ROS-scavenging enzymes in apple leaves under heat stress. **a** In situ accumulation of H_2_O_2_ and superoxide radical (O_2_^−^) before (left panels) and after (right panels) heat treatment revealed by DAB and NBT staining, respectively. Quantitative measurement of **b** H_2_O_2_ and **c** O_2_^−^ concentrations in apple leaves treated with and without high temperature. Activities of **d** superoxide dismutase (SOD), **e** peroxidase (POD) and **f** catalase (CAT) after 4 h of heat treatment. Data are shown as the means of three replicates with SEs. Different letters indicate significant differences between treatments, according to one-way ANOVA and Tukey’s multiple range test (*P* < 0.05).

Functioning as signaling molecules, ROS can regulate plant growth, development, and defense at a low level, and high levels of ROS can cause oxidative damage by disrupting macromolecules and cytomembranes, which results in harmful effects in plants^8^. When stained separately with DAB and NBT, which are used to examine the accumulation of H_2_O_2_ and O_2_^–^, respectively, WT leaves showed more intense blue patches or brown coloration than the leaves of OE plants after heat stress, and the leaves of control plants were similarly and lightly stained (Fig. 2a). This result suggested that under heat stress, the OE plants accumulated less ROS than the WT plants. These results were further confirmed using quantitative measurements (Fig. 2b, c). As the main ROS-scavenging enzymes, SOD converts destructive superoxide radicals into H_2_O_2_, which is still harmful to plants, and POD and CAT can break H_2_O_2_ down immediately into harmless water^27^. Therefore, we measured the activity of these three enzymes and found that they were significantly increased after 4 h of heat treatment and that this increase was enhanced in the transgenic plants (Fig. 2d–f). Together, these results indicated that overexpression of *MdATG18a* enhanced antioxidase activity to reduce the accumulation of toxic ROS.

### Overexpression of MdATG18a in apple improved AsA-GSH cycling under heat stress

**Fig. 3:**
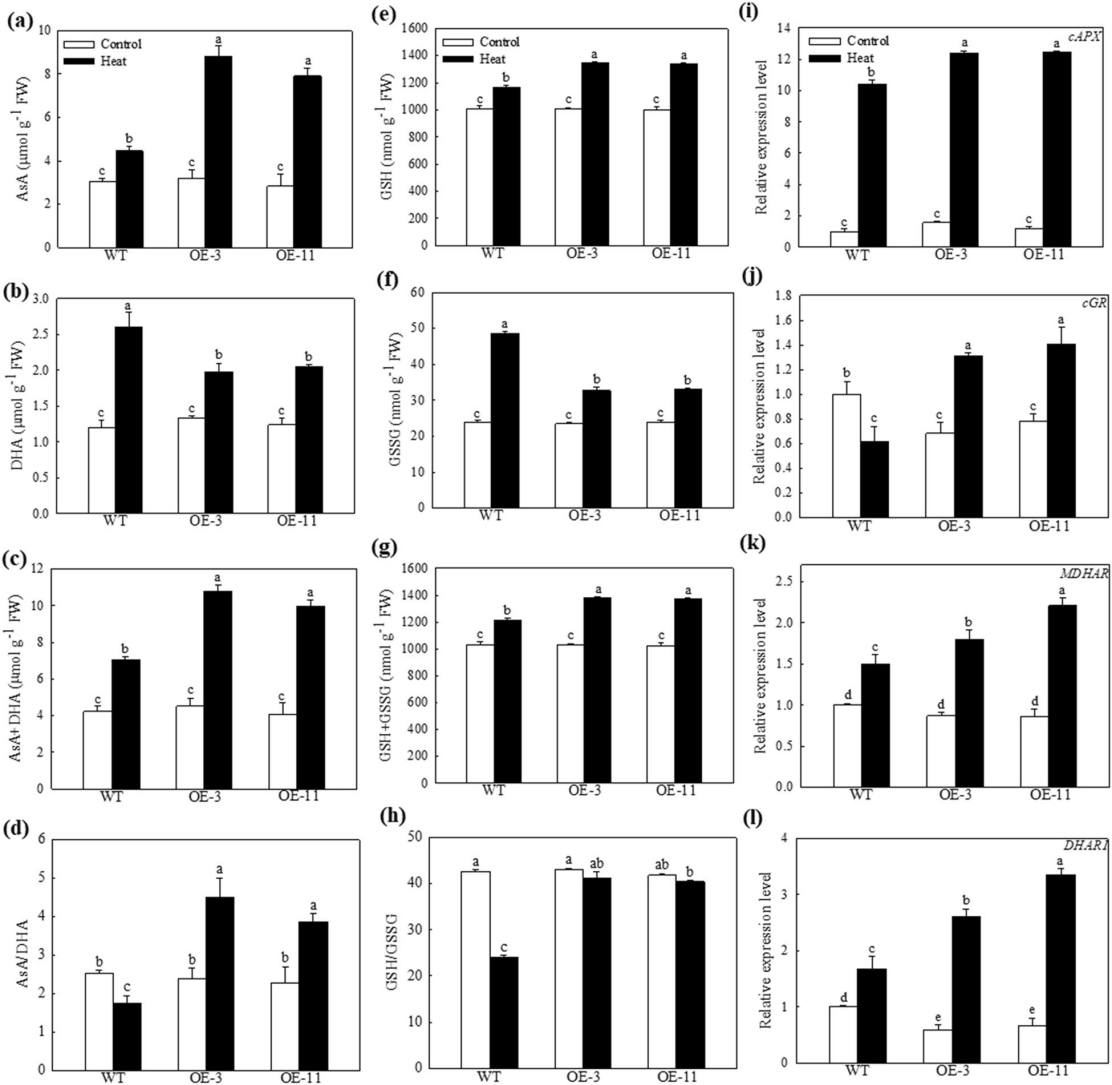
Changes in antioxidant concentrations in apple leaves and the transcript levels of genes implicated in the AsA–GSH cycle under heat stress. Concentrations of **a** ascorbic acid (AsA), **b** dehydroascorbate (DHA) and **c** AsA + DHA and **d** AsA / DHA. Concentrations of **e** glutathione (GSH), **f** oxidized glutathione (GSSG) and **g** GSH + GSSG and **h** GSH/GSSG. These data were measured 4 h after heat treatment. Changes in the expression of **i** *APX*, **j** *GR*, **k** *MDHAR*, and **l** *DHAR1* under heat stress. Total RNA was isolated from leaf samples collected after 2 h of heat stress. Data are shown as the means of three replicates with SEs. Different letters indicate significant differences between treatments, according to one-way ANOVA and Tukey’s multiple range test (*P* < 0.05).

The activity of the AsA-GSH cycle was upregulated in response to the increased production of ROS under high temperature conditions, relieving oxidative stress^28^. We next examined the concentrations of ascorbate and glutathione to further evaluate the regulation of *MdATG18a* in the AsA-GSH cycle. After 4 h of heat treatment, the levels of AsA and DHA were significantly increased, and the total ascorbate concentration was 1.53- and 1.41-fold greater in the OE-3 and OE-11 lines, respectively, than in the WT plants (Fig. 3a–c). This difference was more remarkable when viewed as the ratio of AsA concentration to DHA concentration, and this ratio was diminished in WT plants but increased in the OE lines after heat treatment; no significant difference in this ratio was found in comparison with the control plants (Fig. 3d). The levels of GSH and GSSG, and the total glutathione pool followed a similar pattern (Fig. 3e–g). Meanwhile, the GSH/GSSG ratio declined to a much greater extent in the WT plants than in the transgenic lines under heat stress (Fig. 3h).

We explored changes in the transcript levels of major genes in this cycle. Consistent with changes in the levels and status of AsA and GSH, the transcript levels of *cAPX*, *cGR*, *MDHAR*, and *DHAR1* were increased after 2 h of heat treatment, especially in the transgenic lines (Fig. 3i–l). For example, the expression of *cGR* was 2.13- and 2.28-fold greater in the OE-3 and OE-11 lines, respectively, than in the WT plants after heat stress. These results clearly indicated that overexpression of *MdATG18a* in apple improved its capacity to produce and maintain higher concentrations of beneficial antioxidants under heat stress.

### Heat stress caused less damage to the photosynthetic system in transgenic apple overexpressing *MdATG18a* than in WT apple

**Fig. 4:**
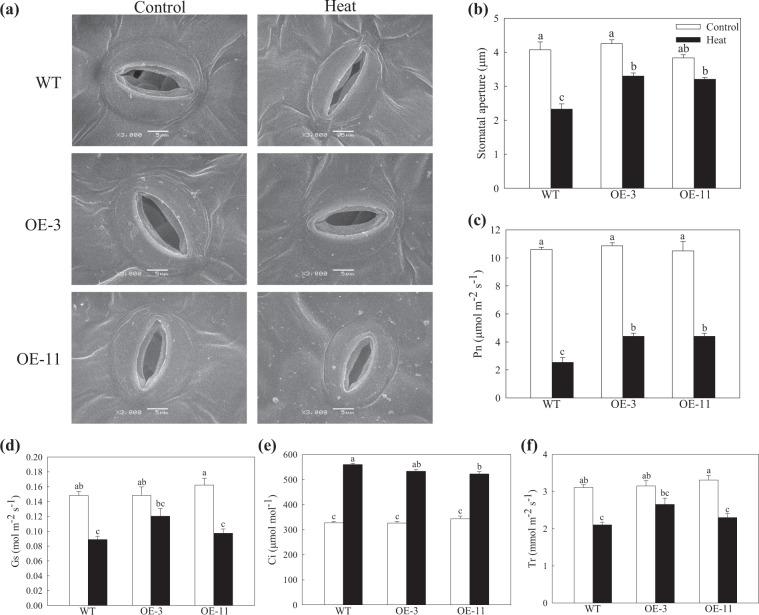
The effect of heat stress on stomatal behavior and the capacity for photosynthesis. **a** Scanning electron microscopy (SEM) images of stomata from plants before (left panels) and after (right panels) 4 h of heat stress. **b** Changes in stomatal apertures under heat stress. Changes in the **c** net photosynthesis rate (Pn), **d** intercellular CO_2_ concentration (Ci), **e** stomatal conductance (Gs), and **f** transpiration rate (Tr) were determined following 18 h of recovery after heat treatment. Data are shown as the means of six replicates with SEs. Different letters indicate significant differences between treatments, according to one-way ANOVA and Tukey’s multiple range test (*P* < 0.05).

Stomatal behavior modulates the transpiration rate and CO_2_ uptake of leaves, both of which strongly influence plant photosynthesis^29^. We observed changes in stomatal morphology between OE lines and WT plants after 4 h of heat treatment. Under normal conditions, there was no difference in stomatal apertures between plants of different genotypes (Fig. 4a, b). High temperature minimized the stomatal aperture, but the degree of shrinkage was decreased in the OE lines.

Photosynthesis is very sensitive to heat stress and can easily be inhibited before other cell functions are impaired^30^. To examine the degree of damage to OE plants in this regard, we measured their gas exchange parameters after an 18-h recovery from heat treatment. Under normal temperature conditions, no difference in Pn, Gs, Ci, or Tr was found between plants of different genotypes. Pn was drastically decreased among plants of all genotypes, but the difference in Pn in the two OE lines was ~1.74-fold greater than that in WT plants (Fig. 4c). Gs and Tr followed a similar trend (Fig. 4d, f). However, Ci increased during the recovery period, but this change was slightly less pronounced in the OE lines (Fig. 4e). These data suggested that the photosynthetic ability of plants overexpressing *MdATG18a* was less damaged than that of WT plants after heat treatment.

### Overexpression of *MdATG18a* in apple influenced photochemical reactions in PSI and PSII under heat stress

**Fig. 5:**
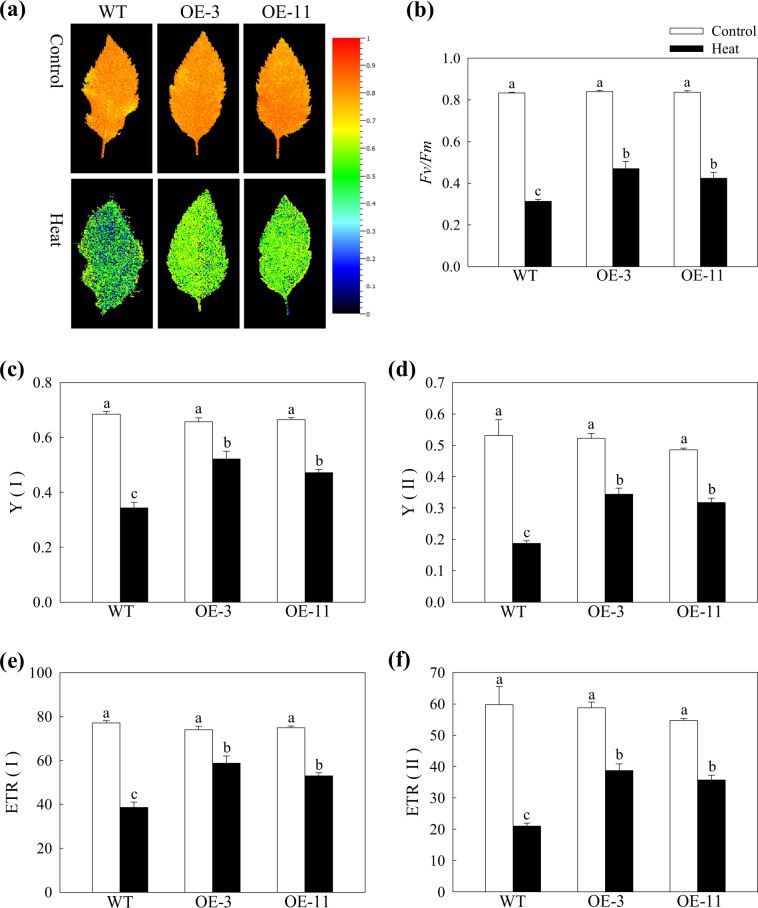
The effect of heat stress on the activity of PSI and PSII in apple leaves. **a** Chlorophyll fluorescence images and **b** *Fv/Fm* ratios of WT and transgenic plants at 25 °C or after 18 h of recovery after heat treatment. The colors in the images indicate *Fv/Fm* ratios ranging from 0 (black) to 1.0 (red). Changes in **c** the effective quantum yield of PSI [Y(I)] and **d** PSII [Y(II)] and **e** the electron transport rate of PSI [ETR (I)] and **f** PSII [ETR (II)]. Data are shown as the means of six replicates with SEs. Different letters indicate significant differences between treatments, according to one-way ANOVA and Tukey’s multiple range test (*P* < 0.05).

Chlorophyll fluorescence measurements have always been used to study the effect of heat stress on photosynthesis in plants^21,31^. We measured the maximum photochemical efficiency of PSII photochemistry (*Fv/Fm*), which represents the amount of absorbed energy trapped in PSII reaction centers^32^. After an 18-h recovery from heat stress, the *Fv/Fm* was significantly decreased by 62.4% in WT plants and by ~50% in the two OE lines (Fig. 5a, b).

To analyze changes in the photochemistry of both PSI and PSII, we used a Dual-PAM-100 measuring system, which simultaneously assesses energy conversion in both PSI and PSII^33^. Heat stress dramatically decreased Y(II) in plants of all genotypes, but Y(II) in the OE-3 and OE-11 lines was 1.84-fold and 1.70-fold greater, respectively, than that in the WT plants (Fig. 5d). ETR(II) followed a similar trend (Fig. 5f). These differences were also reflected in changes in Y(I) and ETR(I) (Fig. 5c, e). Although like Y(II) and ETR(II), Y(I) and ETR(I) were also decreased more in the WT plants, they were inhibited less than Y(II) and ETR(II) by heat stress. Our data suggested that overexpression of *MdATG18a* in apple had a positive effect on photochemical reactions in PSI and PSII after heat stress.

### Overexpression of *MdATG18a* protected chloroplasts in apple from damage under heat stress

**Fig. 6:**
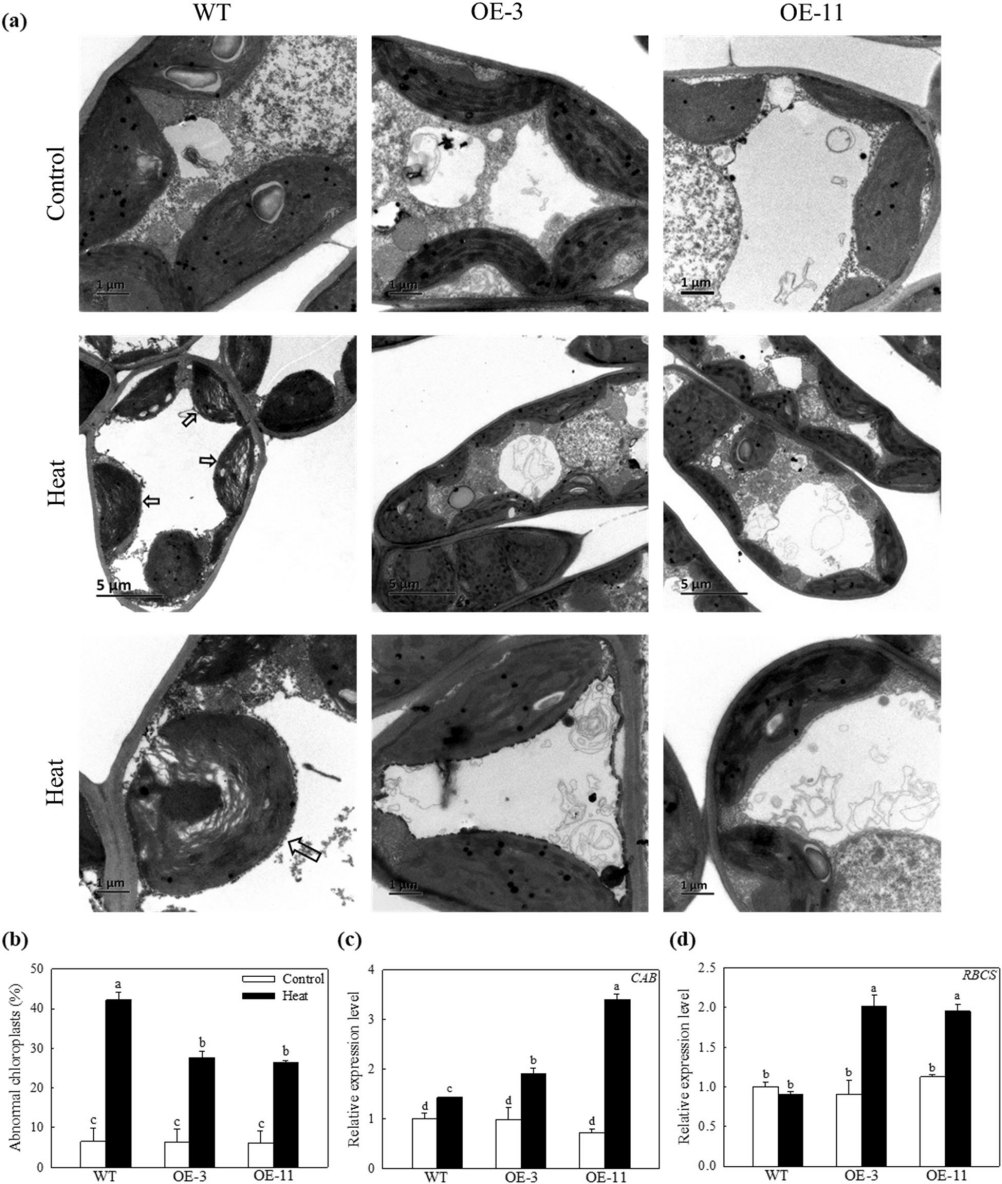
The accumulation of abnormal chloroplasts in the cytoplasm of cells in the heat-exposed leaves of apple plants. **a** TEM images of chloroplasts in mesophyll cells. Chloroplasts that exhibited disorganized thylakoid membranes are indicated by arrows. **b** The proportion of abnormal chloroplasts per cell from experiments described in **a**. Changes in the expression of **c** *MdCAB* and **d** *MdRBCS* under heat stress. Total RNA was isolated from leaf samples collected after 4 h of heat treatment. More than 10 cells were used to quantify chloroplasts. Data are shown as the means of six replicates with SEs. Different letters indicate significant differences between treatments, according to one-way ANOVA and Tukey’s multiple range test (*P* < 0.05).

It is well established that the ultrastructure of chloroplasts and the integrity of thylakoid membranes are extremely sensitive to high-temperature stress^1,34^. Here, we observed changes in the subcellular morphology of chloroplasts in WT and transgenic plants under high temperatures using TEM. The morphology of chloroplasts under normal conditions was normal and indistinguishable among plants of all genotypes (Fig. 6a). However, after 4 h of heat stress, chloroplasts in WT plants had an abnormal morphology with disordered thylakoid membranes or collapsed envelopes, but the chloroplast ultrastructure in the OE lines was less broken and disorganized (Fig. 6a, b). Furthermore, we analyzed changes in the transcript levels of *MdCAB* and *MdRBCS* (Fig. 6c, d), which are related to the activity of chloroplasts under heat stress. Both genes were expressed at higher levels in transgenic lines than in WT plants. The expression of *MdRBCS* was almost 2-fold greater in the OE lines than in WT plants after 4 h of heat stress (Fig. 6d). Overexpression of *MdATG18a* lessened damage to the photosynthetic apparatus housed in the chloroplasts of apple under heat stress.

### Overexpression of *MdATG18a* in apple upregulated the expression of other *MdATG*s and elevated autophagic activity under heat stress

**Fig. 7:**
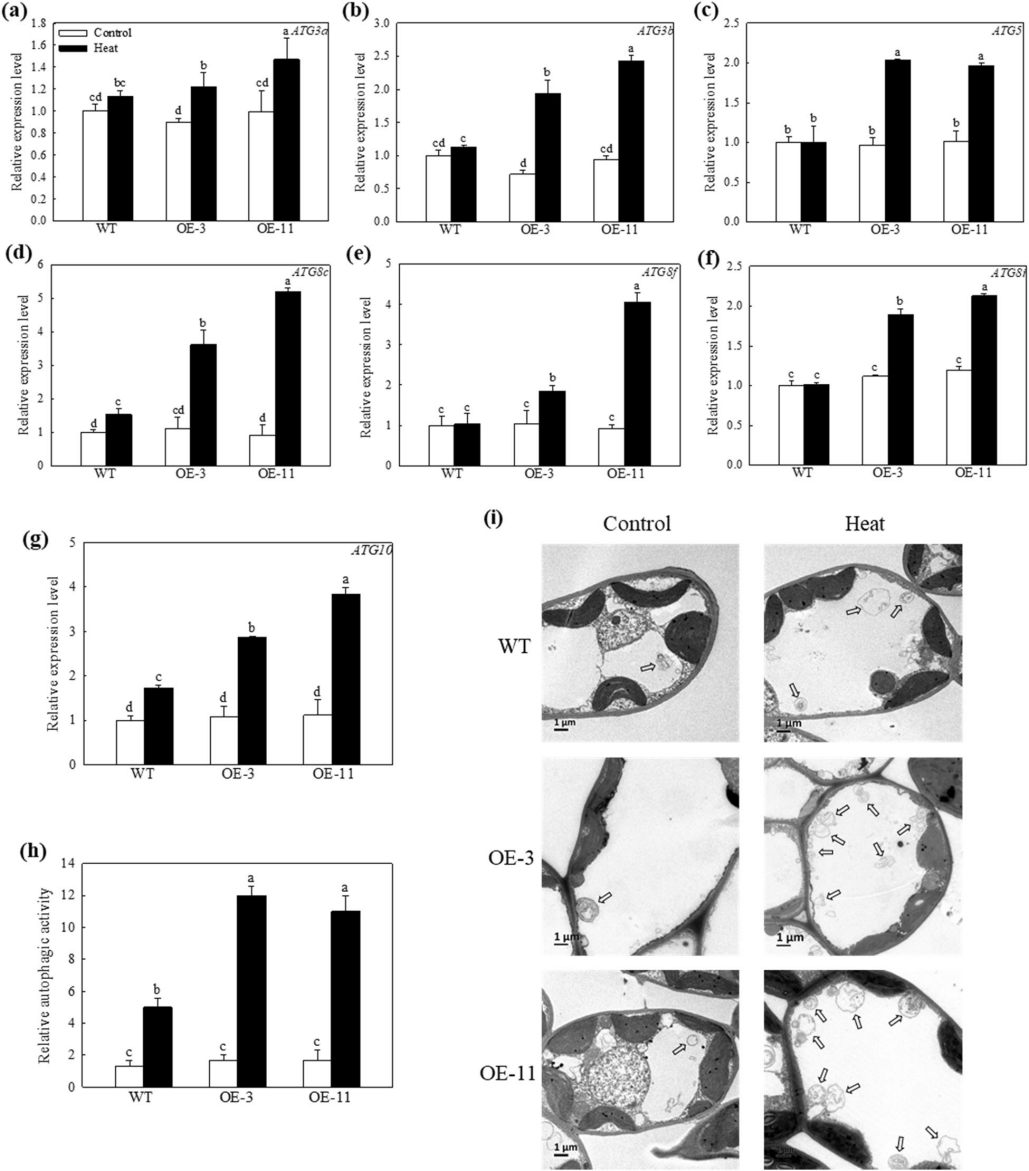
The upregulated expression of several *MdATGs* and accumulation of autophagosomes in apple leaves under heat stress. **a**–**g** Changes in the expression of several *MdATGs* in WT and *MdATG18a*-OE plants after 4 h of heat stress. **h** Representative TEM images of autophagic structures in mesophyll cells from WT and *MdATG18a* OE plants. Autophagosomes are indicated by arrows. Bars: 1 μm. **i** Relative autophagic activity of WT or *MdATG18a*-OE plants shown in **h**. More than 10 cells were used to quantify structures. Data are shown as the means of six replicates with SEs. Different letters indicate significant differences between treatments, according to one-way ANOVA and Tukey’s multiple range test (*P* < 0.05).

To investigate changes in autophagic activity among plants with different genotypes under heat stress, we used qRT-PCR to examine the expression patterns of several important *MdATG*s. The expression of *MdATG3a*, *MdATG3b*, *MdATG5*, *MdATG8c*, *MdATG8f*, *MdATG8i*, and *MdATG10* showed little difference among plants of different genotypes under control conditions (Fig. 7a–g). However, after 4 h of heat treatment, all these tested genes were expressed at higher levels in the OE lines than in WT plants. To further assess changes in autophagic activity among plants with different genotypes under high temperature, we used TEM to observe autophagosome formation in response to heat stress (Fig. 7i). Few autophagosome structures and autophagic bodies were found in all the plants under control conditions, but twice as many autophagosomes and autophagic bodies accumulated in OE lines compared to WT plants under heat stress (Fig. 7h, i). Taken together, these results demonstrated that overexpression of *MdATG18a* significantly promoted the occurrence of autophagy in apple under high temperature.

### Overexpression of *MdATG18a* in apple promoted the expression of *Hsps, HY5*, and *DREB2A* under heat stress

**Fig. 8:**
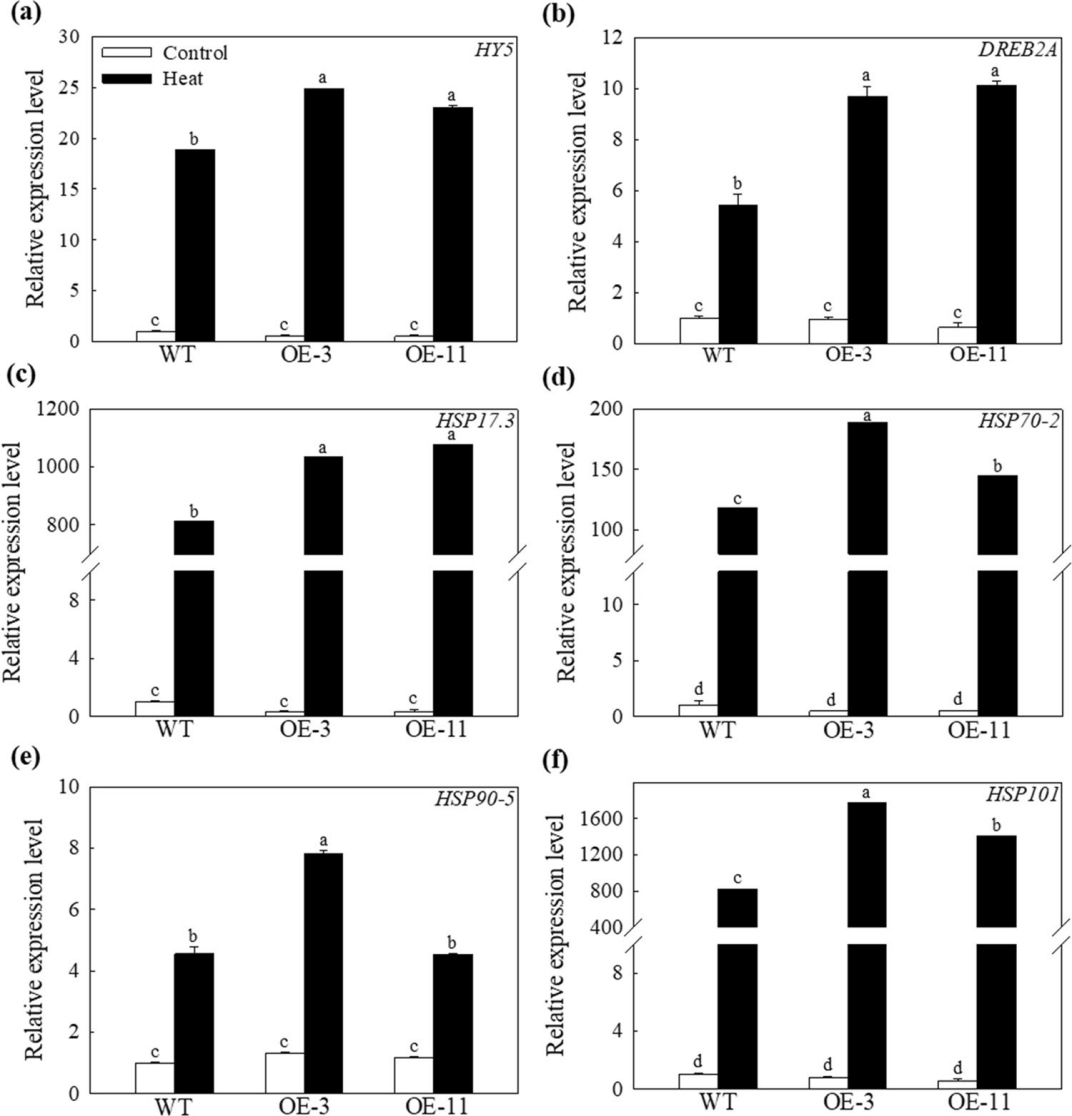
Changes in transcript levels of heat-related genes during stress. Changes in the expression of **a** *MdHY5*, **b** *MdDREB2A*, **c** *MdHSP17.3*, **d** *MdHSP70-2*, **e** *MdHSP90-5*, and **f** *MdHSP101* under heat stress. Total RNA was isolated from leaf samples collected after 2 h of heat stress. Data are shown as the means of six replicates with SEs. Different letters indicate significant differences between treatments, according to one-way ANOVA and Tukey’s multiple range test (*P* < 0.05).

Hsps are induced by heat stress and protect cells from injury by renaturing a variety of heat-denatured proteins^35^. These proteins are divided into five categories in plants based on their approximate molecular weight: Hsp100 proteins, Hsp90 proteins, Hsp70 proteins, Hsp60 proteins, and small heat shock proteins (sHsps)^36^. Here, we measured changes in the expression levels of *MdHsp17.3*, *MdHsp70-2*, *MdHsp90-5*, and *MdHsp101* to identify the potential relationship between *MdATG18a* and *MdHsps*. These four *MdHsps* were greatly upregulated by heat treatment, especially *MdHsp17.3* and *MdHsp101*. Their expression was increased by hundreds or even thousands of times. Additionally, the transcript levels of all four *MdHsps* were much greater in the OE lines than in the WT plants (Fig. 8c–f). Furthermore, we measured changes in the expression of *MdHY5* and *MdDREB2A*, which are important transcription factors in regulating responses to heat stress. *MdHY5* and *MdDREB2A* were also upregulated in transgenic lines under heat stress (Fig. 8a, b). The expression of *MdDREB2A* was 1.79- and 1.87-fold greater in the OE-3 and OE-11 lines, respectively, compared to WT plants after heat stress. These thermal regulation-associated genes crucial for cellular homeostasis were affected by *MdATG18a* under heat stress, which may have increased the tolerance of the transgenic plants to high temperatures.

## Discussion

Autophagy is an evolutionarily conserved protein degradation pathway that balances cellular homeostasis under environmental stress. Several studies indicated that autophagy helps plants alleviate stress due to high temperature. Autophagosomes accumulated in *Arabidopsis* and tomato plants under high temperature stress, and *Arabidopsis atg* mutants and *ATG-*silenced tomato mutants showed higher sensitivity to high temperature than WT plants, accompanied by insoluble protein aggregates^20,21,37^. However, so far, the relationship between autophagy and thermotolerance has been characterized in only model plants, and the underlying physiological mechanisms of autophagy in response to heat stress remain elusive, especially in perennial fruit trees. Therefore, explorations of the functions of ATGs in nonmodal plants, such as apple, will improve our understanding of the various roles of autophagy.

Here, we used apple plants that overexpressed *MdATG18a* to analyze the specific role of *MdATG18a* in response to high temperature (Fig. 1). Our research clearly indicated that overexpression of *MdATG18a* in apple improved tolerance to high temperature. Compared with WT plants, heat stress caused less damage to transgenic plants, which was indicated by decreased electrolyte leakage and MDA and ROS levels and higher RWC levels. Similarly, the decrease in chlorophyll concentration was less pronounced in transgenic apple leaves than in WT apple leaves after heat treatment.

Because the production of excessive ROS is one of the effects of high temperature, several studies have revealed that ROS regulatory systems are crucial for plants in response to heat stress^3,7,38^. For example, ROS metabolic mutants that lacked antioxidant pathways were more deficient in basal thermotolerance^3^. In addition, overexpression of the wheat F-box protein strengthened basal thermotolerance in tobacco, accompanied by higher ROS-scavenging enzyme activity and a lower level of ROS accumulation^39^. We also found that both enzymatic and nonenzymatic mechanisms of ROS scavenging were more active in the *MdATG18a* OE apple lines (Figs. 2 and 3). Upon examination of ROS-scavenging enzymes, we found that the activities of SOD, POD, and CAT were significantly higher in transgenic apple than in WT plants under heat stress. Meanwhile, the total AsA and GSH levels and the ratio of AsA/DHA were elevated in *MdATG18a* transgenic apple under heat stress. Although the GSH/GSSG ratio was slightly decreased in transgenic plants, it was decreased by nearly half in WT plants under heat stress. Consistent with the trend in AsA and GSH concentrations, overexpression of *MdATG18a* induced the expression of *cAPX*, *MDHAR*, *DHAR1*, and *cGR*, which are involved in the AsA–GSH cycle, under heat stress. For example, the GSH/GSSG ratio was decreased by nearly half in WT plants mainly because the expression of *cGR*, which converts GSSG to GSH, was decreased during heat treatment.

High temperature severely affects the photosynthetic efficiency of leaves, primarily due to damage to the mechanisms of photosynthesis and stomatal closure^40^. Extensive evidence supports the conclusion that Rubisco activation, electron transport activity and ATP synthesis are inhibited by moderately elevated temperatures^41,42^. As the temperature rises further above the optimum, the physical integrity of the photosynthetic apparatus is severely disrupted, substantially limiting photosynthesis^43^. In this study, after 18 h of recovery from heat treatment, the Pn decreased drastically among plants of all genotypes, although the reduction was less pronounced in the two OE lines (Fig. 4). Meanwhile, Gs and Tr followed a similar trend. However, Ci increased, while Gs declined during the recovery period, which suggested that the decrease in Pn was not due to a stomatal-limiting factor but was instead due to abnormality in the leaf photosynthetic structure. Changes in stomatal aperture affect transpiration, which is a mechanism of leaf cooling. Therefore, a decrease in stomatal conductance under heat stress leads to an increase in leaf temperature^44^. Furthermore, we found that the shrinkage of stomatal apertures was less pronounced in the OE lines after 4 h of heat treatment, which may have allowed the leaves to maintain a certain transpiration rate to sustain a suitable temperature under heat stress.

Since PSII is the most vulnerable target of heat stress, *Fv/Fm*, photosynthetic electron transfer, and ATP synthesis are often severely damaged under heat stress^45^. The quantum yields of both PSI and PSII were decreased by heat stress^46^. In our study, heat stress decreased the *Fv/Fm* and quantum yields of PSI and PSII among plants of all genotypes (Fig. 5). In general, PSII was more sensitive to high temperature than PSI^47^. For instance, a severe high temperature (43 °C) had no remarkable effect on PSI photochemical capacity in sweet sorghum^48^. However, we found a significant difference in the photochemistry of both PSI and PSII after heat stress between our WT and OE lines; this may have been because the extremely high temperature used (48 °C) caused more irreversible injury to WT plants than to OE lines. All these observations proved that autophagy protects the photosynthetic mechanisms of plants from extreme heat stress.

Similarly, the photosynthetic ability of plants overexpressing *MdATG18a* was less impaired, as reflected in the higher Pn and *Fv/Fm* compared with WT after heat stress. To investigate whether the improved maintenance of photosynthetic ability in OE plants was caused by chloroplast activity, we used TEM to observe changes in the subcellular morphology chloroplasts during heat treatment. Interestingly, we did find that OE lines showed fewer damaged chloroplasts during heat treatment (Fig. 6). Moreover, we also detected stronger autophagic activity in transgenic plants, which was supported by the more pronounced upregulation of other *MdATG* genes and the formation of more autophagosomes in transgenic plants (Fig. 7). Plants have evolved mechanisms to sustain chloroplast function and degrade chloroplasts under certain circumstances. Recent studies have investigated the role of autophagy in the turnover of photodamaged chloroplasts caused by exposure to strong visible light or UV-B^49^ and the way that microautophagy selectively eliminates membrane-damaged chloroplasts^50^. All evidence suggests that autophagy plays an important role in maintaining cell activity by removing damaged chloroplasts under conditions of stress. In addition, because chloroplasts are the main targets of ROS-linked damage in response to heat stress^51,52^, toxic ROS clearance promoted by autophagy also contributes to the protection of chloroplasts under heat stress.

ROS are produced in abundance by photosynthesis in chloroplasts, following which they provide redox signals and function as important regulators of energy and metabolic fluxes^53^. However, damaged chloroplasts are one of the major sites of increased ROS generation during stress, which causes further damage to other macromolecules^13^. Therefore, the timely removal of damaged chloroplasts also reduces ROS-induced damage to plants. In this study, we observed that the overexpression of *MdATG18a* in apple reduced the number of abnormal chloroplasts in mesophyll cells under heat stress, which was probably one reason for the decreased accumulation of toxic ROS in transgenic plants. In addition, we analyzed changes in the transcript levels of *MdCAB* and *MdRBCS*, which are related to the activity of chloroplasts^54^, and found that they were both upregulated more in transgenic lines than in WT plants under heat stress.

*MdATG18a* improved heat resistance by affecting the expression of other heat-related genes because we also detected greater changes in the expression of *MdHY5* and *MdDREB2A*, which can regulate plant responses to heat stress, and changes in four *MdHSPs*, *MdHSP17.3*, *MdHSP70-2*, *MdHSP90-5*, and *MdHSP101*, in OE lines under heat treatment (Fig. 8). In particular, exposure to an extremely high temperature (48 °C) substantially induced the expression levels of *MdHSP17.6* and *MdHSP101* by hundreds or even thousands of times, and they were also upregulated more in the OE lines than in WT plants. Several studies have shown that oxidative stress increased the expression of chloroplast-localized small heat shock proteins^55,56^. In addition, a previous study demonstrated the importance of sHSP26 in protecting maize chloroplasts from heat stress^57^. Thus, these genes probably contributed to the improved performance of plants overexpressing *MdATG18a* under heat stress.

**Fig. 9:**
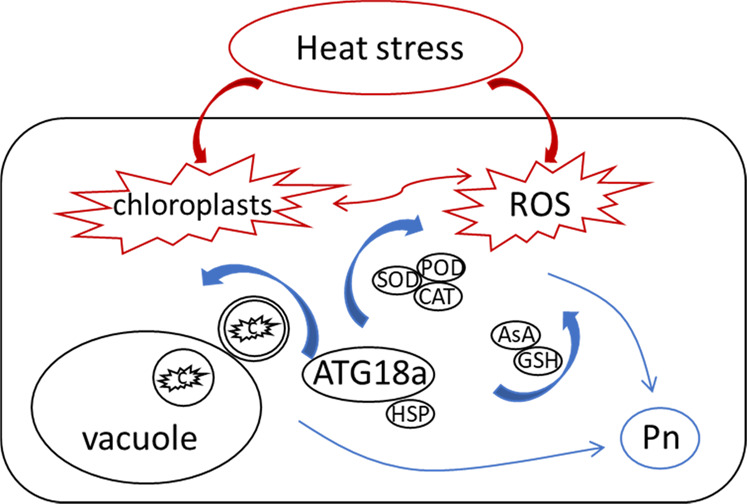
Proposed model of the regulatory function of autophagy in response to heat stress in apple.

In conclusion, our study showed that apple plants overexpressing *MdATG18a* possessed enhanced tolerance to heat stress. Autophagy acts as a core component of heat-responsive signaling and photosynthetic modulation, which are critical pathways of plant thermotolerance (Fig. 9). The exposure of apple overexpressing *MdATG18a* to heat stress led to stronger autophagic activity, which enhanced antioxidant capacity and reduced the accumulation of harmful ROS. In particular, increased autophagic activity led to the increased recycling of damaged chloroplasts during heat treatment, which also reduced the damage to plants due to ROS generated from malfunctioning chloroplasts. In addition, the increased expression of *Hsp* genes was found in OE plants. Therefore, increased autophagic activity decreased heat damage to chloroplasts and caused transgenic plants to exhibit improved photosynthetic capacity after heat stress. These findings provide sufficient physiological and molecular evidence to support the idea that increased autophagic activity increases basal thermotolerance in apple and verify that the integrity of chloroplasts under stress is very important for thermotolerance in apple. Therefore, these findings may have essential applications in horticultural crop breeding in the face of extreme climate change.

## Materials and methods

### Plant materials, growth conditions, and heat treatment

Tissue-cultured GL-3 apple plants were cultivated as described previously, and *MdATG18a* transcript levels were increased by 32- and 36-fold in the OE-3 and OE-11 lines, respectively^23^. After 30 days on rooting media, transgenic and WT plantlets were moved to small plastic bowls (8 × 8 cm) that contained a loam/perlite mixture (1:1, v-v). After adaptation in a growth chamber for 1 month, the seedlings were transferred to medium-sized plastic bowls (12 × 13 cm) and cultivated continuously under conditions of 25 °C, 120 µmol photons m^−2^ s^−1^, and a 14-h photoperiod.

To test their response to high temperature, 2-month-old, healthy plants of uniform size were placed at 48 °C for 6 h in a growth chamber, and plants at a normal temperature (25 °C) were used as the control. At hours 2, 4, and 6 of this experiment, the 3rd-6th leaves from the base of the stems of six plants per treatment were picked. After flash freezing in liquid nitrogen, they were stored at –80 °C.

### RNA extraction and qRT-PCR

Total RNA extraction and cDNA synthesis were separately carried out via a Wolact plant RNA isolation kit (Wolact, Hongkong, China) and a RevertAid First Strand cDNA Synthesis Kit (Thermo Scientific, Waltham, MA, USA) with 1 μg of RNA. qRT-PCR was carried out with a SYBR Premix Ex Taq™ II Kit (Takara, Dalian, China) using a LightCycler 96 quantitative instrument (Roche, Switzerland). *Malate dehydrogenase* (*MDH*) transcription was used to normalize the levels of different genes. Gene-specific primer sequences are shown in the Supplemental Table. All experiments were repeated with three biological replicates, and the relative expression of each gene was determined with the 2^−ΔΔCT^ method^58^.

### Evaluation of stress tolerance

The relative electrolyte leakage (REL) and relative water content (RWC) of the leaves were determined and calculated according to a previously described method^23,59^. Levels of MDA were measured by using Suzhou Comin Biotechnology test kits (Suzhou Comin Biotechnology Co., Ltd, Suzhou, China). Chlorophyll was isolated with 80% acetone, and chlorophyll concentration was detected with a UV-1800 spectrophotometer (Shimadzu, Kyoto, Japan).

### Analysis of the antioxidant system and determination of ROS accumulation

The activities of superoxide dismutase (SOD), peroxidase (POD), and catalase (CAT) and the levels of ascorbic acid (AsA), dehydroascorbate, (DHA), oxidized glutathione (GSSG), glutathione (GSH), H_2_O_2_, and superoxide radical (O_2_^−^) were detected via Suzhou Comin Biotechnology test kits (Suzhou Comin Biotechnology Co., Ltd, Suzhou, China). At the end of the experiment, the presence of H_2_O_2_ and O_2_^−^ in leaf samples was determined via staining with DAB (3,3’-diaminobenzidine) and NBT (nitro blue tetrazolium), respectively.

### Evaluation of photosynthetic characteristics and chlorophyll fluorescence

After recovering for 6 h after the heat treatment, the net photosynthesis rate (Pn), intercellular CO_2_ concentration (Ci), stomatal conductance (Gs), and transpiration rate (Tr) were monitored with a CIRAS-3 portable photosynthesis system (CIRAS, Amesbury, MA, USA). Data were collected from the 4–6th leaves from the base of the stems of eight plants.

Chlorophyll fluorescence transients in the 5th leaves from the base of selected plant stems after 20 min of dark adaptation were measured using Open FluorCam FC 800-O and analyzed with Fluorcam7 software (PSI, Brno, Czech Republic)^60^.

### Observations of leaf stomata, chloroplasts, and autophagosomes

After 4 h under experimental conditions, mature leaves at the same position from each selected plant were excised and immediately cut into small pieces. Then, they were fixed with a 4% glutaraldehyde solution and maintained at 4 °C for 12 h. Stomata on the leaves were observed with a JSM-6360LV scanning electron microscope (SEM; JEOL Ltd., Tokyo, Japan) as previously described^61^. Chloroplasts and autophagosomes were observed on a JEOL-1230 transmission electron microscope (TEM, Hitachi, Japan) as described before^23,49^.

### Measurements of the quantum yields of PSI and PSII

Energy conversion and electron transport in PSI [Y(I)] and PSII [Y(II)] in apple leaves were measured synchronously using a Dual-PAM-100 system (Heinz Walz, Effeltrich, Germany) according to Fluo C P700 mode as previously described^62^.

### Statistical analysis

Experimental data were analyzed with SPSS 20.0 software. One-way ANOVA was used to compare significant differences based on the Tukey’s multiple range test (*P* < 0.05), and values are represented as the means ± SEs (standard errors).

## Supplementary information

Primers used in this study

## References

[1] Allakhverdiev SI (2008). Heat stress: an overview of molecular responses in photosynthesis. Photosynth. Res..

[2] Schoffl F, Prandl R, Reindl A (1998). Regulation of the heat-shock response. Plant Physiol..

[3] Larkindale J, Hall JD, Knight MR, Vierling E (2005). Heat stress phenotypes of arabidopsis mutants implicate multiple signaling pathways in the acquisition of thermotolerance. Plant Physiol..

[4] Suzuki N, Bajad S, Shuman J, Shulaev V, Mittler R (2008). The transcriptional co-activator MBF1c is a key regulator of thermotolerance in *Arabidopsis thaliana*. J. Biol. Chem..

[5] Sung D, Kaplan F, Lee KJ, Guy CL (2003). Acquired tolerance to temperature extremes. Trends Plant Sci..

[6] Li B, Gao K, Ren H, Tang W (2018). Molecular mechanisms governing plant responses to high temperatures. J. Integr. Plant Biol..

[7] Choudhury FK, Rivero RM, Blumwald E, Mittler R (2017). Reactive oxygen species, abiotic stress and stress combination. Plant J..

[8] Mittler R (2011). ROS signaling: the new wave?. Trends Plant Sci..

[9] Mittler R, Vanderauwera S, Gollery M, Van Breusegem F (2004). Reactive oxygen gene network of plants. Trends Plant Sci..

[10] Chen B, Retzlaff M, Roos T, Frydman J (2011). Cellular strategies of protein quality control. Cold Spring Harb. Perspect. Biol..

[11] Larkindale J, Vierling E (2008). Core genome responses involved in acclimation to high temperature. Plant Physiol..

[12] Miller G (2007). Double mutants deficient in cytosolic and thylakoid ascorbate peroxidase reveal a complex mode of interaction between reactive oxygen species, plant development, and response to abiotic stresses. Plant Physiol..

[13] Wang Q, Chen J, He N, Guo F (2018). Metabolic reprogramming in chloroplasts under heat stress in Plants. Int. J. Mol. Sci..

[14] Sharkey TD (2005). Effects of moderate heat stress on photosynthesis: importance of thylakoid reactions, rubisco deactivation, reactive oxygen species, and thermotolerance provided by isoprene. Plant Cell Environ..

[15] Yamamoto Y (2008). Quality control of photosystem II: impact of light and heat stresses. Photosynth. Res..

[16] Mittler R, Finka A, Goloubinoff P (2012). How do plants feel the heat?. Trends Biochem. Sci..

[17] Shah F (2011). Impact of high-temperature stress on rice plant and its traits related to tolerance. J. Agric. Sci..

[18] Deng Y (2011). Heat induces the splicing by *IRE1* of a mRNA encoding a transcription factor involved in the unfolded protein response in *Arabidopsis*. Proc. Natl Acad. Sci. USA.

[19] Avin-Wittenberg T (2018). Autophagy-related approaches for improving nutrient use efficiency and crop yield protection. J. Exp. Bot..

[20] Zhou J (2013). NBR1-Mediated Selective autophagy targets insoluble ubiquitinated protein aggregates in plant stress responses. PLoS Genet..

[21] Zhou J, Wang J, Yu J, Chen Z (2014). Role and regulation of autophagy in heat stress responses of tomato plants. Front. Plant Sci..

[22] Sedaghatmehr M (2019). A regulatory role of autophagy for resetting the memory of heat stress in plants. Plant Cell Environ..

[23] Sun X (2018). Improvement of drought tolerance by overexpressing *MdATG18a* is mediated by modified antioxidant system and activated autophagy in transgenic apple. Plant Biotechnol. J..

[24] Sun X (2018). *MdATG18a* overexpression improves tolerance to nitrogen deficiency and regulates anthocyanin accumulation through increased autophagy in transgenic apple. Plant Cell Environ..

[25] Sun X (2018). Overexpression of *MdATG18a* in apple improves resistance to Diplocarpon mali infection by enhancing antioxidant activity and salicylic acid levels. Hortic. Res..

[26] Wang P (2014). Isolation and characterization of *MdATG18* alpha, a WD40-repeat AuTophaGy-related gene responsive to leaf senescence and abiotic stress in Malus. Sci. Hortic..

[27] Xu Y, Burgess P, Zhang X, Huang B (2016). Enhancing cytokinin synthesis by overexpressing ipt alleviated drought inhibition of root growth through activating ROS-scavenging systems in *Agrostis stolonifera*. J. Exp. Bot..

[28] Ma Y (2008). Effects of high temperature on activities and gene expression of enzymes involved in ascorbate-glutathione cycle in apple leaves. Plant Sci..

[29] Hu LY (2018). Exogenous myo-inositol alleviates salinity-induced stress in *Malus hupehensis Rehd*. Plant Physiol. Biochem..

[30] Mathur S, Agrawal D, Jajoo A (2014). Photosynthesis: response to high temperature stress. J. Photochem. Photobiol. B Biol..

[31] Wang X, Xu C, Cai X, Wang Q, Dai S (2017). Heat-responsive photosynthetic and signaling pathways in plants: insight from proteomics. Int. J. Mol. Sci..

[32] Nakamura S, Izumi M (2018). Regulation of chlorophagy during photoinhibition and senescence: lessons from mitophagy. Plant Cell Physiol..

[33] Porcar-Castell A (2014). Linking chlorophyll a fluorescence to photosynthesis for remote sensing applications: mechanisms and challenges. J. Exp. Bot..

[34] Semenova GA (2004). Structural reorganization of thylakoid systems in response to heat treatment. Photosynthetica.

[35] Yang M (2017). Identification of *MsHsp20* gene family in *Malus sieversii* and functional characterization of *MsHsp16.9* in heat tolerance. Front. Plant Sci..

[36] Hu W, Hu G, Han B (2009). Genome-wide survey and expression profiling of heat shock proteins and heat shock factors revealed overlapped and stress specific response under abiotic stresses in rice. Plant Sci..

[37] Yang X, Srivastava R, Howell SH, Bassham DC (2016). Activation of autophagy by unfolded proteins during endoplasmic reticulum stress. Plant J..

[38] de Pinto MC, Locato V, Paradiso A, De Gara L (2015). Role of redox homeostasis in thermo-tolerance under a climate change scenario. Ann. Bot..

[39] Li Q (2018). Wheat F-box protein gene *TaFBA1* is involved in plant tolerance to heat stress. Front. Plant Sci..

[40] Wise RR, Olson AJ, Schrader SM, Sharkey TD (2004). Electron transport is the functional limitation of photosynthesis in field-grown Pima cotton plants at high temperature. Plant Cell Environ..

[41] Carmo-Silva AE, Salvucci ME (2011). The activity of Rubisco’s molecular chaperone, Rubisco activase, in leaf extracts. Photosynth. Res..

[42] Schrader SM, Wise RR, Wacholtz WF, Ort DR, Sharkey TD (2004). Thylakoid membrane responses to moderately high leaf temperature in Pima cotton. Plant Cell Environ..

[43] Salvucci ME, Crafts-Brandner SJ (2004). Relationship between the heat tolerance of photosynthesis and the thermal stability of rubisco activase in plants from contrasting thermal environments. Plant Physiol..

[44] Fauset S (2019). Contrasting responses of stomatal conductance and photosynthetic capacity to warming and elevated CO_2_ in the tropical tree species *Alchomea glandulosa* under heatwave conditions. Environ. Exp. Bot..

[45] Havaux M (1993). Characterization of thermal-damage to the photosynthetic electron-transport system in potato leaves. Plant Sci..

[46] Ahammed GJ, Xu W, Liu A, Chen S (2018). *COMT1* silencing aggravates heat stress-induced reduction in photosynthesis by decreasing chlorophyll content, photosystem II activity, and electron transport efficiency in tomato. Front. Plant Sci..

[47] Li H (2016). Unraveling main limiting sites of photosynthesis under below- and above-ground heat stress in cucumber and the alleviatory role of luffa rootstock. Front. Plant Sci..

[48] Yan K (2013). Dissection of photosynthetic electron transport process in sweet sorghum under heat stress. PLoS ONE.

[49] Izumi M, Ishida H, Nakamura S, Hidema J (2017). Entire photodamaged chloroplasts are transported to the central vacuole by autophagy. Plant Cell.

[50] Nakamura, S., Hidema, J., Sakamoto, W., Ishida, H. & Izumi, M. Selective elimination of membrane-damaged chloroplasts via microautophagy. *Plant Physiol*. **177**, 1007–1026 (2018). 10.1104/pp.18.00444 PMC6052986 29748433

[51] Asada K (2006). Production and scavenging of reactive oxygen species in chloroplasts and their functions. Plant Physiol..

[52] Komayama K (2007). Quality control of photosystem II: Cleavage and aggregation of heat-damaged D1 protein in spinach thylakoids. Biochim. Biophys. Acta-Bioenerg..

[53] Foyer CH (2018). Reactive oxygen species, oxidative signaling and the regulation of photosynthesis. Environ. Exp. Bot..

[54] Wang P (2014). Melatonin regulates proteomic changes during leaf senescence in *Malus hupehensis*. J. Pineal Res..

[55] Kim KH (2012). Overexpression of a chloroplast-localized small heat shock protein OsHSP26 confers enhanced tolerance against oxidative and heat stresses in tall fescue. Biotechnol. Lett..

[56] Lee BH (2000). Expression of the chloroplast-localized small heat shock protein by oxidative stress in rice. Gene.

[57] Hu X (2015). Protein sHSP26 improves chloroplast performance under heat stress by interacting with specific chloroplast proteins in maize (*Zea mays*). J. Proteom..

[58] Livak KJ, Schmittgen TD (2001). Analysis of relative gene expression data using real-time quantitative PCR and the 2^−^^Δ^^ΔCT^ method. Methods.

[59] Dionisio-Sese ML, Tobita S (1998). Antioxidant responses of rice seedlings to salinity stress. Plant Sci..

[60] Perez-Bueno ML, Pineda M, Diaz-Casado E, Baron M (2015). Spatial and temporal dynamics of primary and secondary metabolism in *Phaseolus vulgaris* challenged by *Pseudomonas syringae*. Physiol. Plant..

[61] Liang B (2018). Effects of exogenous dopamine on the uptake, transport, and resorption of apple ionome under moderate drought. Front. Plant Sci..

[62] Deng C, Zhang D, Pan X, Chang F, Wang S (2013). Toxic effects of mercury on PSI and PSII activities, membrane potential and transthylakoid proton gradient in *Microsorium pteropus*. J. Photochem. Photobiol. B Biol..

