# Supplementary material for: MdATG18a overexpression improves basal thermotolerance in transgenic apple by decreasing damage to chloroplasts
Source: Hortic Res. 2020 Mar 1;7:21. doi: 10.1038/s41438-020-0243-2 (PMC7049305; doi:10.1038/s41438-020-0243-2)
Supplement: Supplementary file 1 — Primers used in this study [file 41438_2020_243_MOESM1_ESM.pdf]

Supplemental Table Primers used in this study

**Gene**	**Sequence (5'-3')**	**Purpose**
MDH	F: CGTGATTGGGTACTTGGAAC<br>R: TGGCAAGTGACTGGGAATGA	Reference gene used in real-time PCR
q*cAPX*	F: AACTACAAGGGATGAAGCC<br>R: CAACGAGGATGATAACCAG	Quantitative expression of *MdcAPX*
q*cGR*	F: GTTCAGCGACAAGGCGTAT<br>R: TCAACCGATTTCCATTTCC	Quantitative expression of *MdcGR*
q*MDHAR*	F: CCATACTTCTATTCCCGCTCCT<br>R: CGACCACCTTCCCGTCTTT	Quantitative expression of *MdMDHAR*
q*DHAR1*	F: AGTGGACGGTTCCAGCAGA<br>R: TTCCCATCCCGCAATCAC	Quantitative expression of *MdDHAR1*
q*HY5*	F: AACTAGCATCCAAAGCTAGCC<br>R: CCACAGTCTGCAGTTTCACAAG	Quantitative expression of *MdHY5*
q*DREB2A*	F: CAGAGAACTTCACAACCGAC<br>R: CCAGTGCTCATGGCAAGGTTTT	Quantitative expression of *MdDREB2A*
q*HSP17.3*	F: AAGTTTGTGTTGCCGGAG<br>R: ATGTTATAATCCCTGCACGA	Quantitative expression of *MdHSP17.3*
q*HSP70-2*	F: CTCTGTCTCTCGGTTTGGAG<br>R: GTCTTGTCCTCGGCAGAAA	Quantitative expression of *MdHSP70-2*
q*HSP90-5*	F: ACACTCACTTCACCACTGAG<br>R: CATTTGAATCCACCACACCC	Quantitative expression of *MdHSP90-5*
q*HSP101*	F: CTATTCGGAGATGGCTGGAG<br>R: TGATGGAGGAAAGGAATT	Quantitative expression of *MdHSP101*
q*CAB*	F: ATCTTGATGGGTGCCGTGGAG<br>R: AGCCTCTGTGTCTTCTGCAAGG	Quantitative expression of *MdCAB*
q*RBCS*	F: AAACTGGGTTCCCTGCTTGGA<br>R: GGCAGCTTCCACATTGTCCAG	Quantitative expression of *MdRBCS*
q*ATG3a*	F: AAGGGGGCGGAGATGGTTC<br>R: GCACTTAGAGACGAGGTTATCGC	Quantitative expression of *MdATG3a*
qATG3b	F: AGGGAGATGGTTTTGAAACAGA<br>R: ACTTAGAGACGAGGTTATCGC	Quantitative expression of *MdATG3b*
q*ATG5*	F: GCAGGTCGTGTTCCAGTTC<br>R: CCTCCTCCTCCTTGTATCTCAA	Quantitative expression of *MdATG5*
q*ATG8c*	F: GCGTTCAAGATGGAGCACCCTC<br>R: CAGCCCTTTCCACAACCACTGG	Quantitative expression of *MdATG8c*
q*ATG8f*	F: TCGTAGACAATGTCCTCCCAGC<br>R: CCAAATGTGTTCTCGCCACTGT	Quantitative expression of *MdATG8f*
q*ATG8i*	F: GCAGCAGGCTTCACTTGACTCC<br>R: GGAATCCATGCGACTGGCTGTT	Quantitative expression of *MdATG8i*
q*ATG10*	F: TGGAACCAGCGAGTGGATGAAG<br>R: ACAACTGAGAGCCAAGACACCA	Quantitative expression of *MdATG10*
